# Sealing Wax and Bottles in Bags—A Paradigm Shift in Refined Olive Oil Packaging: Preliminary Results

**DOI:** 10.3390/foods12224161

**Published:** 2023-11-17

**Authors:** Monica Macaluso, Nicola Mercanti, Giulio Scappaticci, Elena Lannocca, Linda Rossi, Luca Guidi, Paolo Tondello, Francesco Brazzarola, Angela Zinnai

**Affiliations:** 1 Department of Agriculture, Food and Environment, University of Pisa, Via del Borghetto 80, 56124 Pisa, Italy; monica.macaluso@unipi.it (M.M.); giulio.scappaticci@phd.unipi.it (G.S.); e.lannocca@studenti.unipi.it (E.L.); angela.zinnai@unipi.it (A.Z.); 2 Salov, S.p.A., Via di Montramito, 1600, 55054 Massarosa, Italy; linda.rossi@salov.com (L.R.); luca.guidi@salov.com (L.G.); 3 FT System S.r.l., Via Leonardo da Vinci, 117, 29010 Alseno, Italy; paolo.tondello@ftsystem.com (P.T.); francesco.brazzarola@antaresvision.com (F.B.); 4 Interdepartmental Research Centre “Nutraceuticals and Food for Health”, University of Pisa, Via del Borghetto 80, 56124 Pisa, Italy

**Keywords:** refined olive oil, storage, packaging, laser spectroscopy

## Abstract

Generally, olive oil possesses natural protection against oxidation due to antioxidant compounds such as phenols and tocopherols. However, in the case of refined olive oil, the refining process unavoidably reduces the presence of these compounds. Considering these considerations, the objective of this study was to address the issues related to the “tightness” of the cap used for packaging oil in SALOV, aiming to extend the product’s shelf life. The oil under investigation was packaged in 250 mL transparent glass bottles, each filled with either argon or air. Subsequently, the samples were divided into three groups: one group sealed with a conventional screw cap, another covered with a special protective bag, and a third one sealed with a wax cover directly on the cap. The storage period varied, during which the atmospheric conditions were monitored daily through both destructive and non-destructive analyses. The preliminary results indicate that alternative preservation techniques, such as the use of argon, sealing wax, and protective bags, can effectively enhance the shelf life of the oil and maintain its quality (reduce oxidation, preserve phenolic compounds, and reduce the degradation of pigments). Further research and development in this area could lead to the production of high-quality extra virgin olive oils with extended shelf life and improved sensory and nutritional properties.

## 1. Introduction

The preservation of food products plays a crucial role in determining their quality [1]. Consequently, there is an increasing focus on studying new techniques or enhancing existing ones to ensure the preservation of food and the production of safe, high-quality food products [2,3]. Oxygen is one of the factors that significantly affect the shelf life of oil [4]. It plays a role in the degradation processes of both saponifiable and unsaponifiable fractions, which are responsible for the beneficial and nutraceutical properties associated with olive oil [5]. Olive oil, in general, possesses a natural defense against oxidation provided by antioxidant compounds such as phenols and tocopherols [6,7,8,9]. However, in the case of refined oil, the effective concentrations of these compounds are reduced due to the refining process [10,11,12,13,14,15,16]. Packaging also plays a vital role in maintaining food quality throughout the supply chain [17,18]. It helps preserve hygienic requirements, prevents foodborne illnesses, and facilitates the movement of food from production to consumers. Modern packaging materials should not only fulfill these functions but also aim to minimize their impact on the environment, addressing the long-term challenge of plastic waste disposal. Furthermore, sustainable food consumption requires reducing the environmental footprint of packaged food [19]. Plastics are often perceived as having a significant environmental impact, mainly due to their end-of-life perspective, disregarding considerations of material recyclability and the production and transportation impacts of packaging materials [17,18,20]. In light of these considerations, the present work had the objective of solving the problems related to the “tightness” of the caps used in a refining factory for packaging oil, with a consequent increase in the shelf life of the product. The oil tested was packaged in 250 mL transparent glass bottles (with argon and air, respectively) and then subsequently divided into three lots: one closed with a classic screw cap, one covered with a special protective bag, and one by a sealing wax cover directly on the cap. The experimentation envisaged conservation for a period of four months, monitoring the atmospheres daily (through destructive and non-destructive analyses) in order to verify the “tightness of the corks” and of the additional variables (sealing wax and protective bags). Finally, at regular intervals of 30 days, product and quality destructive analyses were carried out to verify the effects of conservation on the chemical parameters of the oil. This study represents a preliminary investigation in which we have used an experimental approach to evaluate the possible application of this type of packaging: it may be profitably used in other sectors, such as in wine and bakery products, it is easy to find, and it is simple to apply in the industrial field, as it does not imply modifications of the bottling line.

## 2. Materials and Methods

### 2.1. Raw Material

For the experimentation, refined olive oil produced by the SALOV company was used. The oil was produced following an enzymatic refining process, according to the protocol used in the company, as previously reported [5]. The oil obtained was automatically bottled directly from the tank, using an in-line bottling machine using gas.

Before being packaged, the necessary parameters required to act as a control in the analyses and to evaluate the effectiveness of the conservation methods were evaluated.

### 2.2. Experimental Plan

The refined oil was packaged in 250 mL transparent glass bottles, each containing 200 g of oil, and closed with a screw cap. Then, 72 small bottles were obtained; these were divided into two parts and packaged, respectively, in air and in argon.

The bottles were subsequently packaged using three different methods:

1. Only with the screw cap;
2. Adding a layer of sealing wax on the cap;
3. By placing the bottle inside a protective bag (filled with air and argon, respectively).

At the end of the packaging, the following were obtained:

- Twelve bottles with screw caps packed in argon;
- Twelve bottles with a layer of sealing wax packed in argon;
- Twelve bottles inside a protective bag (filled with argon) packed in argon;
- Twelve bottles with screw caps packed in air;
- Twelve bottles with a layer of sealing wax packed in air;
- Twelve bottles inside a protective bag (filled with air) packed in air.

The experimentation envisaged storage for a period of 4 months, monitoring the atmospheres daily (through non-destructive analyses, thanks to FT-SYSTEM machinery) in order to judge the tightness of the caps and additional methods (sealing wax and protective bag). At approximately 30-day intervals, product and quality destructive analyses were conducted to verify the effects of storage on the chemical parameters of the oil.

### 2.3. Packaging Composition

Table 1 shows the specific characteristics of each material used. In particular, regarding the sealing wax are reported color, solubility, wax content, viscosity, purity grade, moisture content and melting point; otherwise, regarding the bag, are reported type of structure, coupled layer, type, suitable type, layer thickness, weight, total weight and the water value permeability.

### 2.4. Destructive Analysis

#### 2.4.1. Legal Quality Parameters

According to the EU Regulation No. (UE) 2022/2104 [21], the FFA and the PV determinations, as well as the specific absorbances at 232 and 270 nm (K232, K270, and ΔK) of the ROOs, were carried out in duplicate.

#### 2.4.2. Total Phenol Determination

Total phenols were extracted following the procedure of Servili et al. [22] with slight modifications [19], and extracts were stored at −20 °C under N_2_ atmosphere until use. The total phenol concentration of the refined oil was determined calorimetrically at 765 nm using the Folin–Ciocalteu reagent [19], and calculations were carried out using a calibration curve with gallic acid as a standard.

#### 2.4.3. Chlorophylls and Carotenoids

Chlorophylls and carotenoids were determined at 670 nm and 470 nm, respectively, following Minguez-Mosquera et al.’s [23] protocol. The oil samples were dissolved in cyclohexane (1.5:5 *w*/*v*), and absorbance was measured using a Perkin Helmer Lambda 10 UV–vis spectrophotometer.

Chlorophylls = (A_670_ × 106)/(613 × 100 × d) (1)

Carotenoids = (A_470_ × 106)/(2000 × 100 × d) (2)

### 2.5. Non-Destructive Analysis

To evaluate the composition of the atmosphere present in the head space of the bottles and inside the bags, two machines designed and developed by the FT SYSTEM were used. These two instruments allowed for a simple and fast test without sample preparation.

For the evaluation of the atmosphere inside the bags, EVO-P was used to determine the content of CO_2_ and O_2_ (for this study’s thesis, we focused on the oxygen value). The technology that the instrument uses employs two monochromatic UV lasers, calibrated to the specific frequency of the gases under examination, which pass through the sample and are then measured at the outlet; on the basis of the variation that the intensity of the laser light undergoes during its passage due to absorption, the concentration of the specific gas is measured.

Instead, EVO-1O2 was used to control the atmospheres in the head space of the bottles, in which a very low-power laser beam crosses the space. The sent signal is adjusted in wavelength and amplitude in order to interact with the gaseous molecules. The received signal is also, in this case, modified by the interaction with the gas present in the space, and this allows for a quantification of the oxygen present internally in the space.

### 2.6. Statistical Analysis

The results are the means ± SD of two/three independent experiments. The significance of differences among means was determined by one-way ANOVA (CoStat, Cohort 6 software, Monterey, CA, USA). Comparisons among means were performed by Bartlett’s X2 corrected test (*p* < 0.05). PCA (principal component analysis) was performed using JMP software, Cary, NC, USA [24]. The boxplot was made using Python 3.10 software, Auckland, New Zealand.

## 3. Results and Discussion

### 3.1. Characterization of the Refined Oil

At the beginning of the experimentation, the refined oil used had a very low acidity value and reduced values both for the number of peroxide and for the spectrophotometric indices. Considering the values of the qualitative parameters (total phenols, bitterness intensity, chlorophylls, and carotenoids), the results obtained showed a characteristic refined oil characterized by a fairly low content of bioactive compounds [5] (Table 2).

Immediately after bottling, the first measurements of the atmospheres were carried out (Table 3) in order to evaluate their trend during the storage period (4 months) and the effect of using the protective gas (in the case of argon) in preserving the quality of the product [19].

### 3.2. Atmosphere Trend during Storage

**Figure 1:**
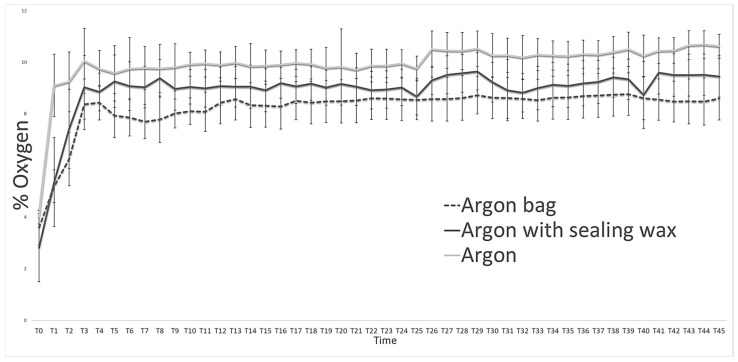
Storage atmospheres (argon) trend during the entire observation period in the three types of packages used (naked bottle, bottle with wax, and bag). Data are reported as the mean of the values with *n* = 10.

Throughout the experimentation period, the storage atmospheres and their trends were evaluated (Figure 1) using the machinery developed by FT SYSTEM, both for checking the bare bottles and for checking the bottles stored inside the bag. From Figure 1, we can see how the use of sealing wax better preserves the protective atmosphere inside the bottle, considering the daily monitoring carried out on the three types of packaging used (Table 1). However, if we consider the inert atmosphere “actually” present inside the bottles (Figure 1), the quantity of oxygen present inside the bottle packaged with the protective bag is considerably lower than all the other theses considered.

### 3.3. Trend of Chemical Parameters during Storage

#### 3.3.1. Free Fatty Acidity, Peroxides Value, and Spectrophotometric Indices

At regular intervals of 30 days, the destructive chemical analyses were carried out (acidity, amount of peroxide, spectrophotometric indices, total phenols, chlorophylls, and carotenoids) throughout the entire storage period.

**Figure 2:**
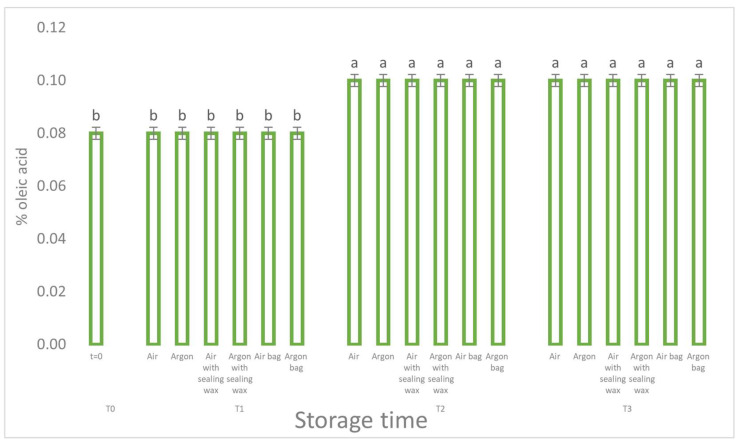
Free acidity trend as a function of storage time (t = 0, t = 1, t = 2, t = 3) for the 6 samples considered. Letters (a, b) indicate significant differences (*p* < 5%) over time after ANOVA (analysis of variance).

Analyzing the results, we can see how the free acidity values (Figure 2), an indicative parameter of the hydrolytic process for the different packaging methods, did not report any statistically significant differences. The increase in free acidity is, in fact, the same for all the theses considered.

**Figure 3:**
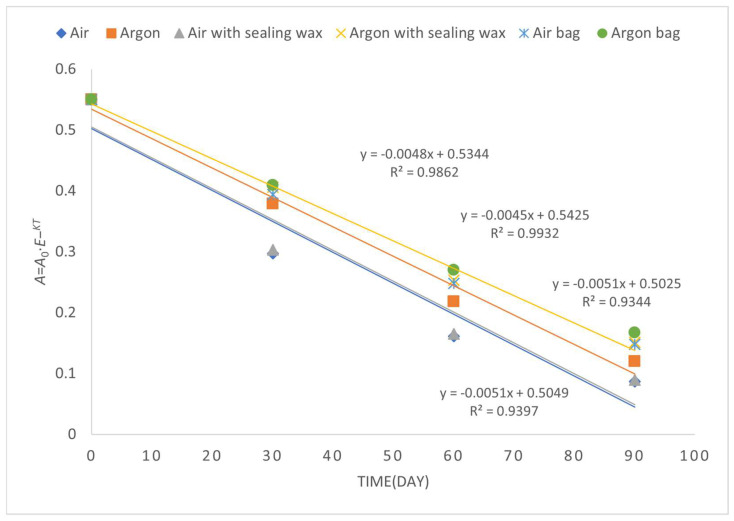
First-order kinetic constant related to the time evolution of peroxide value over a period of 90 days of storage.

However, the differences are evident by observing the degradation kinetics of the peroxide value (Figure 3), which are more contained in the samples preserved in argon with the addition of sealing wax and, above all, the bag, which, in fact, turns out to be the sample with the lowest value (1.6 meqO_2_).

**Figure 4:**
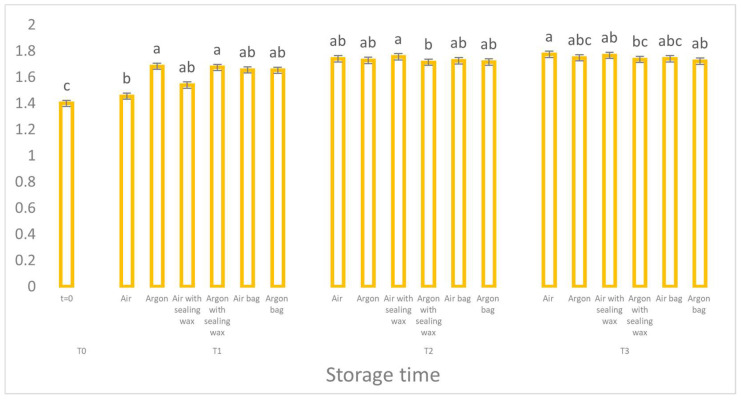
Trend of K232 values as a function of storage time (t = 0, t = 1, t = 2, t = 3) for the 6 samples considered. Letters (a, b, etc.) indicate significant differences (*p* < 5%) over time after ANOVA (analysis of variance).

During the whole observation period, the peroxide values increased (Figure 4) following a first-order kinetic equation (Equation (3)):

[A]_t = t_ = [A]_t = 0_ · e^−kt^ (3)

where

[A]_t = t_ = Peroxide value at a generic time (t = t).

[A]_t = 0_ = Peroxide value at the start of the observation period (t = 0).

*k* = first-order kinetic constant (days − 1).

*t* = time (days).

Afterward, the kinetic constant was calculated following Equation (4):

(4) $$k=\frac{-ln(\frac{A}{A_{0}})}{t}$$

In Table 4, the medium kinetic rate constants (*k*) for different conditions of preserving refined olive oil are provided. The kinetic rate constants represent the rate coefficients of the chemical reaction and reflect the rate of oil deterioration under different packaging conditions [19].

Analyzing the data, we can observe some interesting trends. In general, the use of argon as an inert gas appears to have a positive effect on oil preservation in comparison to air [19]. The kinetic rate constants for oil preserved in an argon atmosphere (Argon) are lower than those for oil preserved in air (Air), indicating a slower rate of deterioration.

Furthermore, the addition of a sealing wax cover on the cap (Air with sealing wax and Argon with sealing wax) seems to provide an additional benefit in oil preservation compared to conditions without sealing wax. The kinetic rate constants for oil with sealing wax are lower than those without sealing wax, indicating greater stability of the oil over time.

Finally, the use of a protective bag (Air bag and Argon bag) appears to have the best significant impact on oil preservation compared to the other conditions. The kinetic rate constants for oil with the protective bag are lower than those without the bag.

Overall, the data highlight the importance of different packaging conditions in preserving refined olive oil. The use of inert gases like argon and the addition of a sealing wax and bag for the cover can contribute to greater stability of the oil, reducing the rate of deterioration. However, it is important to note that the effectiveness of different packaging conditions may also depend on other factors, such as the initial quality of the oil and overall storage conditions.

The value of K232 (Figure 4), used together with peroxide as a marker of the primary oxidative process, confirms a significantly reduced oxidative trend in the samples preserved in argon, although the statistical difference between the samples is not particularly high.

**Figure 5:**
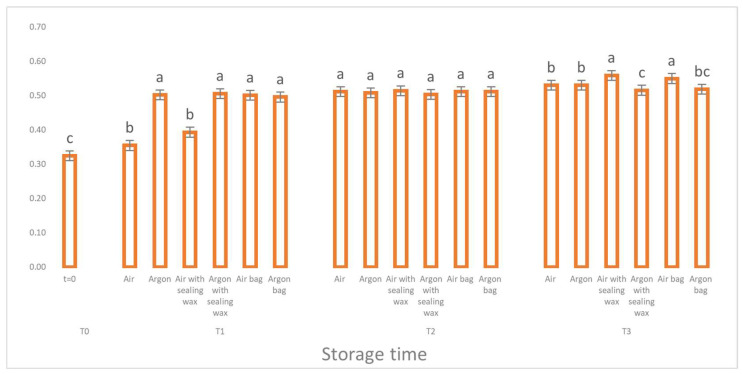
Trend of K270 values as a function of storage time (t = 0, t = 1, t = 2, t = 3) for the 6 samples considered. Letters (a, b, etc.) indicate significant differences (*p* < 5%) over time after ANOVA (analysis of variance).

Also, for the K270 parameter (Figure 5), relating to the absorption of secondary oxidation compounds (unsaturated carbonyl compounds), we can see how the samples packaged in argon with the addition of the external bag and the sealing wax layer have lower values compared to their equivalents which were stored in the air. However, all the samples show values higher than 0.25 (the limit set for virgin olive oils), denoting the presence of a refined oil.

#### 3.3.2. Total Phenols, Chlorophylls, and Carotenoids

**Figure 6:**
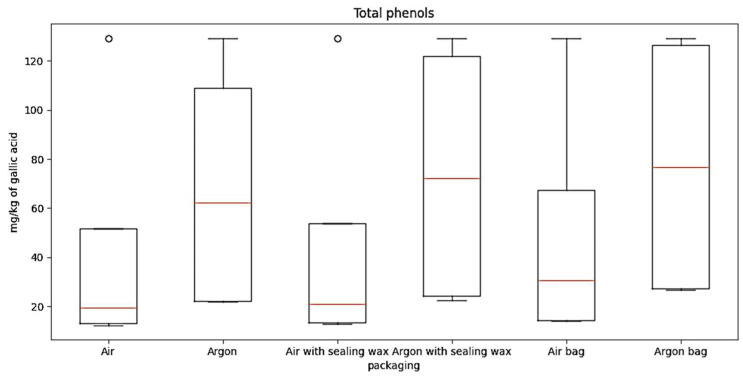
The boxplot for total phenols for the six packaging combinations considered.

Regarding total phenols data, processed through a boxplot (Figure 6), it is possible to observe the following information:

“Air” condition: The median is around 20, indicating a moderate concentration of phenols.

Interquartile range (IQR): The IQR extends from approximately 15 to 46, representing the variation in the middle 50% of the data.

“Argon” condition: The median is around 60, indicating a significantly higher concentration compared to the “Air” condition.

IQR: The IQR extends from approximately 22 to 125, indicating a higher variation in the data compared to the “Air” condition.

“Air with sealing wax” condition: The median is around 20, indicating a moderate concentration of oil.

IQR: The IQR extends from approximately 13 to 50, representing a moderate variation in the data.

“Argon with sealing wax” condition: The median is around 63, indicating a significantly higher concentration of oil.

IQR: The IQR extends from approximately 22 to 119, representing a considerable variation in the data.

“Air bag” condition: The median is around 23, indicating a relatively low concentration of oil.

IQR: The IQR extends from approximately 15 to 60, indicating a moderate variation in the data.

“Argon bag” condition: The median is around 65, indicating a significantly higher concentration of oil.

IQR: The IQR extends from approximately 22 to 120, representing a relatively small variation in the data.

Overall, the boxplot provides an overview of the oil concentration distribution for each storage condition. It can be observed that conditions with argon generally have higher concentrations compared to their respective conditions without argon.

**Figure 7:**
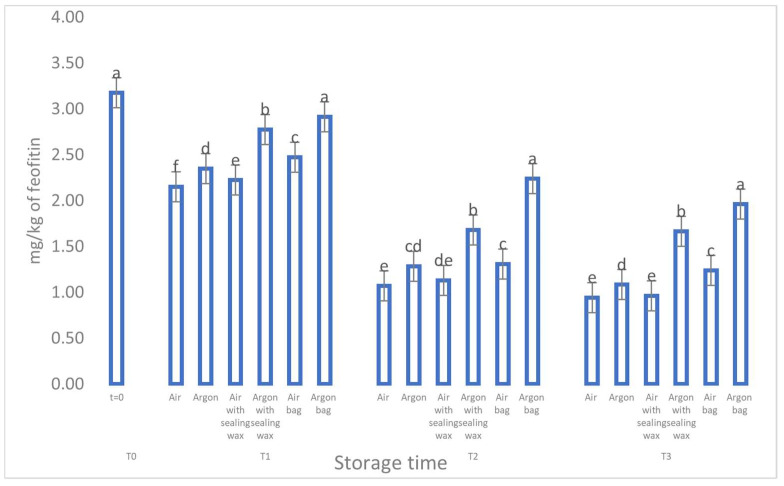
Trend of sample chlorophylls as a function of storage time (t = 0, t = 1, t = 2, t = 3) for the 6 samples considered. Letters (a, b, etc.) indicate significant differences (*p* < 5%) over time after ANOVA (analysis of variance).

**Figure 8:**
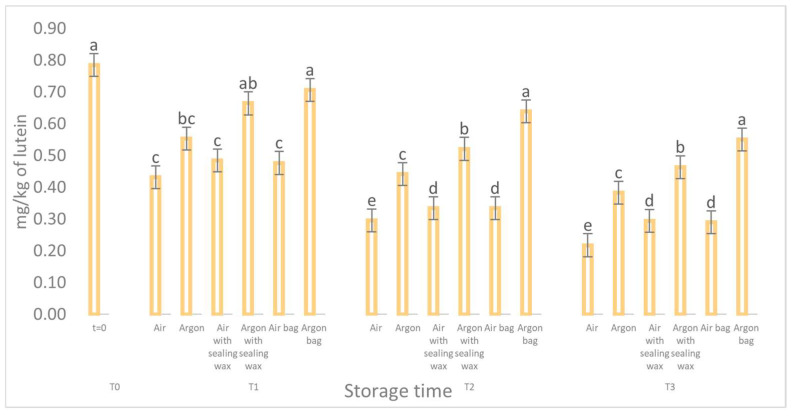
Trend of the carotenoids of the samples as a function of the storage time (t = 0, t = 1, t = 2, t = 3) for the 6 samples considered. Letters (a, b, etc.) indicate significant differences (*p* < 5%) over time after ANOVA (analysis of variance).

The data concerning the chlorophyll and carotenoid content (as shown in Figure 7 and Figure 8) and their correlation to the presence of pheophytin and lutein, respectively [23], reaffirm the pattern observed for the phenolic compounds. During storage, all analyzed samples exhibited a consistent and significant reduction in these compounds, particularly the samples stored in air. However, oils stored in argon with the additional sealing wax and an external bag better preserved these compounds and maintained higher values throughout the entire preservation period.

### 3.4. Principal Component Analysis (PCA)

**Figure 9:**
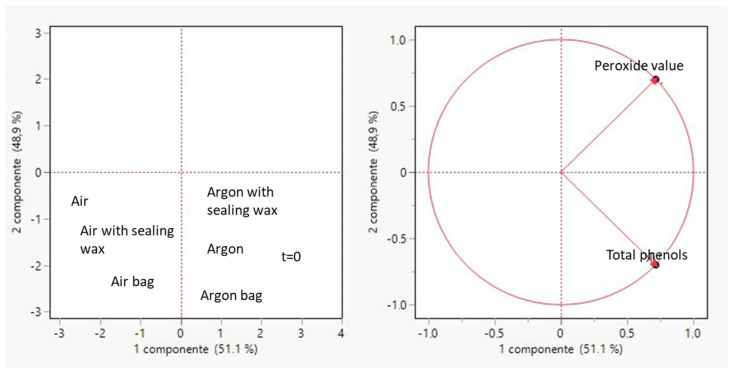
Principal component analysis (PCA) performed in order to correlate peroxide values with total phenols for all considered samples.

The principal component analysis was performed in order to correlate the course of the degradation processes with the decrease of phenolic compounds. From Figure 9, it can be seen that the three types of packaging used are arranged in the same quadrant, highlighting a similar trend in the degradative processes, which lead to an inevitable degradation of the phenolic compounds as previously described [25]. Specifically, the presence of argon sealed within the bottle enables the product to resist oxidation for an extended period. At the initial moment (time zero), the oil occupies the same category as the three oils packaged in the three different types of containers; however, this is achieved by incorporating argon into the packaging process.

## 4. Conclusions

In conclusion, the results indicate that alternative preservation techniques, such as the use of argon, sealing wax, and protective bags, can effectively enhance the shelf life of the oil and maintain its quality. These methods demonstrated the ability to reduce oxidation, preserve phenolic compounds, and minimize the degradation of pigments. Further research and development in this area could lead to the production of high-quality extra virgin olive oils with extended shelf life and improved sensory and nutritional properties.

These methods could also be applied to already existing packaging lines, thanks to very small (and also inexpensive) modifications that allow for considerable advantages over time.

## Figures and Tables

**Table 1:** Characterization of the sealing wax and bag used for the experimentation.

**Sealing Wax Characteristics**	
**APPEARANCE**	Semi-transparent flakes
**COLOR**	Amber yellow and reddish brown
**SOLUBILITY**	Soluble in alcohol, esters, ketones, in acidic or alkaline
**WAX CONTENT**	4.5%
**VISCOSITY**	(Ford Cup No. 4—20 °C—50% Ethanol Solution) 80 s
**PURITY GRADE**	Arsenic max 2 ppm Heavy metals max 10 ppm Insoluble substances in ethanol, varying percentage depending on the type (max. 2% for anhydrous powder type) Other resins (Rosin, Copal, etc.) absent
**MOISTURE CONTENT**	(Karl Fischer Method) max. 5%
**MELTING POINT**	70–75 °C
**Bag Characteristics**	
**TYPE OF STRUCTURE**	Coupled/multilayer
**COUPLED LAYER**	Polypropylene
**TYPE**	Coextruded bioriented
**SUITABLE TYPE**	Vibac CT
**LAYER THICKNESS**	20 microns
**WEIGHT**	18.2 g/mq
**COUPLED LAYER**	Polypropylene
**LAYER THICKNESS**	25 microns
**WEIGHT**	22.7 g/mq
**TOTAL WEIGHT**	45.0 g/mq
**WATER VALUE PERMEABILITY (38 °C/90% UR)**	5.0 g/mq 24 h

**Table 2:** Characterization of the refined oil at the first time (t = 0).

Chemical Parameters	
Free fatty acidity (%) oleic acid	0.08 ± 0.01
Peroxide value (meqO_2_/Kg of oil)	0.55 ± 0.12
K_232_	1.41 ± 0.01
K_270_	0.32 ± 0.01
Total phenols (mg/kg of gallic acid)	174.21 ± 0.28
Chlorophylls (mg/kg of feofitin)	3.17 ± 0.10
Carotenoids (mg/kg of lutein)	0.88 ± 0.04

**Table 3:** Initial values of the atmospheres.

Samples	% Oxygen
Air	19.24 ± 0.21
Air with sealing wax	18.41 ± 0.32
Air Bag	16.78 ± 0.28
Argon	3.60 ± 0.55
Argon with sealing wax	3.43 ± 0.41
Argon Bag	3.52 ± 0.25

**Table 4:** First-order kinetic constant related to the time evolution of peroxide value over the end of storage phase.

First-Order Kinetic Constant ± i.c			
Air	0.74 ± 0.05	Argon	0.45 ± 0.03
Air with sealing wax	0.53 ± 0.04	Argon with sealing wax	0.42 ± 0.02
Air bag	0.43 ± 0.02	Argon bag	0.37 ± 0.02

## Data Availability

The data presented in this study are available on request from the corresponding author.
